# Dr. Oliver H. Babcock

**Published:** 1911-09

**Authors:** 


Dr. Oliver H. Babcock, a graduate of the University of Buffalo, of the class of 1861, after practising in Hammondsport, N. Y. for nearly fifty years, died July 29, 1911, aged 77.

